# Biochemical properties of glycerol kinase from the hypersaline-adapted archaeon *Haloferax volcanii*

**DOI:** 10.1128/aem.00886-25

**Published:** 2025-07-08

**Authors:** Karol M. Sanchez, Julie A. Maupin-Furlow

**Affiliations:** 1 Department of Microbiology and Cell Science, Institute of Food and Agricultural Sciences, University of Florida 53701, Gainesville, Florida, USA; 2 Genetics Institute, University of Florida 145773, Gainesville, Florida, USA; Kyoto University, Kyoto, Japan

**Keywords:** glycerol kinase, halophilic archaea, haloarchaea, subunit structure, enzyme optimization, kinetic analysis, metabolism, biocatalyst

## Abstract

Extremophilic microorganisms are promising candidates for industrial and analytical biocatalysis. *Haloferax volcanii*, a halophilic archaeon that prefers glycerol over glucose, channels this substrate into central metabolism through glycerol kinase (GK). Here, we report the biochemical properties of *H. volcanii* GK and its potential for biotechnological applications. An N-terminal His-tagged GK was functional *in vivo* and yielded 3 mg/L culture—4.5 times more enzyme than a C-terminal StrepII-tagged version. Size exclusion chromatography revealed a glycerol-induced oligomeric shift from homodimer to a dimer-dominant state with detectable tetramer. The purified enzyme showed robust activity across broad pH and salinity ranges, with optimal activity at 100 mM NaCl and 50°C–60°C. It retained catalytic activity in 5%–10% dimethyl sulfoxide (DMSO) and crude glycerol containing methanol. His-GK was freeze-thaw stable and thermotolerant in 2 M NaCl buffers. In the absence of ligands, the enzyme’s melting temperature (T_m_) was 80°C. Glycerol increased the T_m_ to 85°C, and combinations with MgCl₂ (84°C) or ATP (88°C) provided further stabilization. The highest T_m_ (89°C) occurred with all three ligands, suggesting a cumulative stabilizing effect. Kinetic analyses revealed positive cooperativity for glycerol, ATP, and Mg²^+^; Mn²^+^ and Co²^+^ also supported the activity. *H. volcanii* GK is the first known GK to exhibit positive cooperativity with glycerol and ATP. Its high stability and substrate flexibility support its use in biodiesel waste valorization, *in vitro* biocatalysis, and biosensor development—applications demanding robust, specific, and stable enzymes.

**IMPORTANCE:**

This study reveals that *H. volcanii* GK exhibits positive cooperativity for glycerol, ATP, and Mg²^+^, a kinetic feature not previously reported for glycerol kinases. This behavior enables steep, switch-like responses to small substrate changes, offering unique advantages for biosensor design. Importantly, *H. volcanii* GK also maintains high activity under extreme salinity, temperature, broad pH, and solvent conditions that typically limit enzyme use in industrial and environmental applications. These traits make this GK an ideal candidate for enzyme-based biosensors, which often suffer from poor tolerance to pH, solvent, and thermal stress. Its robustness supports its use in cross-linked enzyme crystals, an immobilization method that enhances enzyme stability and reusability under harsh conditions. Moreover, GKs are already employed in Mg²^+^ detection kits; however, *H. volcanii* GK’s ability to tolerate and respond to diverse divalent cations (e.g., Co²^+^, Mn²^+^) broadens their potential for pollutant detection and environmental monitoring. These features collectively position *H. volcanii* GK as a valuable biocatalyst for biosensing, *in vitro* diagnostics, and biotechnological applications requiring both precision and durability.

## INTRODUCTION

Glycerol is an abundant carbon source with the potential to be used in numerous applications in the cosmetic, pharmaceutical, food, and renewable chemical industries (1). While the biodiesel industry produces large quantities of glycerol as a byproduct, approximately one pound per gallon of biodiesel (2), this excessive accumulation poses challenges due to its high volume, limited market applications, and environmental concerns (2, 3). However, glycerol waste streams also provide opportunities to transform this three-carbon intermediate into valuable chemicals such as 1,3-propanediol, butanol, ethanol, biodiesel, biomethane, monoglycerides, citric acid, and docosahexaenoic acid (2, 4, 5). Beyond waste valorization, glycerol can also play a key role in *in vitro* biocatalysis and biosensor development.

Enzyme-based biosensors are increasingly employed in clinical diagnostics and environmental monitoring, yet their performance is frequently hindered by poor solvent tolerance, limited thermal stability, and narrow pH operating windows (6). In this context, GK is a particularly attractive enzymatic component, as it catalyzes a well-defined, ATP-dependent reaction with potential for precise signal generation. Purified GKs have been integrated into biosensor systems for real-time monitoring of magnesium ions (Mg²^+^), such as during the degradation of magnesium implants (7). In addition, the use of GK in commercial assay kits (e.g., Sigma-Aldrich Cat. No. MAK026) to quantify Mg²^+^ concentrations underscores its practical diagnostic value. However, current systems lack robustness under harsh or mixed-metal conditions, limiting broader application.

These limitations create a niche for GKs with enhanced biochemical stability and dynamic control features. In particular, positive cooperativity, where substrate binding enhances further substrate affinity, offers opportunities to engineer biosensors with steep, threshold-like responses (8). Such digital-like switching behavior enhances sensitivity and enables detection of low-abundance analytes with higher precision. While cooperative kinetics are widely used in systems biology to achieve tunable outputs, they have not been observed in previously characterized GKs.

Purified enzymes are widely used in biosensors, analytical assays, and cell-free biocatalysis, where regulatory compliance and stability under diverse conditions (e.g*.*, high glycerol, temperature, pH, dimethyl sulfoxide [DMSO]) are crucial for performance, specificity, and reproducibility (9–11). Such applications highlight GK’s broad versatility, offering new opportunities for *in vitro* biocatalysis, environmental monitoring, and the development of diagnostic platforms in biomedicine and beyond. These applications require robust microorganisms that display a preference for glycerol as a carbon source and enzymes capable of functioning efficiently under extreme conditions (2, 4).

The hypersaline-adapted archaeon *Haloferax volcanii*, originally isolated from the Dead Sea (12), has garnered attention based on its robust growth in harsh conditions and preference for glycerol as its primary energy and carbon source (13–17). Other organisms from the Dead Sea, such as the algae *Dunaliella*, use a “salt-out” strategy that relies upon counterbalancing the osmotic stress through production of organic osmolytes, such as glycerol, which can leak out into the environment for use by the halophilic archaea (18). Thus, *H. volcanii* has unusual properties, not found in most organisms, including the preference for glycerol over glucose, and an entire proteome adapted with halophilic, organic solvent-tolerant, and thermophilic properties, including surfaces reduced in hydrophobicity and enriched in acidic residues compared to mesohalic counterparts (19–22). These adaptations make haloarchaeal enzymes attractive not only for industrial processes that require stability under harsh conditions but elevated for advanced applications such as *in vitro* biocatalysis, biosensor integration, and real-time diagnostics, where enzyme performance in non-conventional environments is critical (9). Moreover, *H. volcanii* transcription factors, GlpR, TrmB, TbsP, and GfcR, are identified to regulate central metabolic pathways, offering valuable tools for redirecting metabolic fluxes through synthetic biology strategies (23–26). The combination of halophilic properties, environmental resilience, and specialized carbon utilization pathways positions this archaeon as a promising platform for a broad range of industrial and biotechnological innovations (14).

Glycerol kinase (GK) plays a central role in glycerol metabolism. In *H. volcanii*, glycerol is metabolized through the action of GK and glycerol-3-phosphate (G3P) dehydrogenase to generate 1,3-dihydroxyacetone phosphate (DHAP) for central metabolism (13, 27). Using ATP, GK phosphorylates intracellular glycerol to form G3P and ADP as a byproduct. This metabolic step is essential for glycerol consumption, as GK-deficient mutants are unable to grow on glycerol compared to other carbon sources (13). Optimizing the purification and characterizing the biocatalytic properties of haloarchaeal GKs are crucial for advancing fundamental knowledge and broadening their applicability across the bioindustry, including *in vitro* biocatalysis, biosensor development, diagnostic platforms, and biodiesel waste valorization.

Here we report the purification and biochemical characterization of a haloarchaeal GK. This study focused on *H. volcanii* GK, as it represents a promising candidate for the biotransformation of glycerol in diverse biotechnological settings. We find that the *H. volcanii* GK displays resilient properties and provides the first example of a GK that exhibits positive cooperativity for glycerol, ATP, and magnesium. The *H. volcanii* GK is observed to undergo a glycerol-induced conformational change from dimer to a tetramer consistent with its apparent positive allosteric control by glycerol. Thus, the *H. volcanii* GK is found to be fundamentally distinct from characterized GKs, expanding our understanding of extremophilic enzymes and their potential for applications such as *in vitro* biocatalysis, biosensor development, and environmental or clinical diagnostics, in addition to glycerol bioconversion in biodiesel-related processes. As our understanding of archaeal metabolism grows, research on *H. volcanii* continues to contribute valuable insights and tools to the broader landscape of synthetic biology and enzyme-based technologies.

## MATERIALS AND METHODS

### Strains and media

Strains and plasmids utilized in this study are listed in Table 1. *Escherichia coli* strain Top10 (Life Technologies, Carlsbad, CA) was used for routine cloning procedures. To obtain plasmids devoid of methylation for subsequent transformation into *Haloferax volcanii*, *E. coli* strain GM2163 (New England Biolabs, Ipswich, MA) was employed, following the protocol described by Cline et al*.* (28). Liquid cultures were aerated by orbital shaking at 200 rpm. *E. coli* strains were cultured at 37°C in Luria-Bertani (LB) medium, with ampicillin (100 mg·L⁻¹) added when required. *H. volcanii* strains were grown at 42°C in American Type Culture Collection 974 rich medium (ATCC974) or minimal medium supplemented with 20 mM glycerol (GlyMM) or fructose (FMM). Media was prepared according to the protocols in *The Halohandbook* (29). Growth media supplements included novobiocin (0.2 µg·mL⁻¹), 5-fluoroorotic acid (5-FOA, 50 µg·mL⁻¹), and uracil (50 µg·mL⁻¹). Before adding to the medium, 5-FOA was dissolved in 100% DMSO at 50 mg·mL⁻¹.

**TABLE 1:** List of strains and plasmids used in this study^*a*^

Strain or plasmid	Description	Source or reference
*E. coli* strains		
Top 10	F– *recA1 endA1 hsdR17(r_K_–m_K_+) supE44 thi-1 gyrA relA1*	Invitrogen
GM2163	F– *ara-14 leuB6 fhuA31 lacY1 tsx78 glnV44 galK2 galT22 mcrA dcm-6 hisG4 rfbD1rpsL136 dam13::*Tn*9 xylA5 mtl-1 thi-1 mcrB1 hsdR2*	New England Biolabs
*H. volcanii* strains		
H26	DS70 *ΔpyrE2*	(30)
H1207	*ΔpyrE2 pitA_Nph_ Δmrr*	(31)
KS4	H26 *ΔglpK*	(13)
KM01	KS4 *pitA_Nph_*	This study
Plasmids		
pTA131	Ap^r^; pBluescript II containing P*fdx*:*pyrE2*	(30)
pJAM202c	Ap^r^ Nv^r^; control plasmid derived from pBAP5010	(32)
pTA1106	Ap^r^; pTA131 with *pitA_Nph_* gene replacement	(31)
pJAM2666	Ap^r^ Nv^r^; pJAM2055 with P2*_rrn_-glpK-strepII* (expressing GK-StrepII)	(13)
pJAM503	Ap^r^ Nv^r^; pBAP5010 with P2*_rrn_-His6-panA*	(32)
pJAM4351	Ap^r^ Nv^r^; pJAM503 derived with P2*_rrn_-his6-glpK* (expressing His-GK)	This study

^*a*^ Ap^r^, ampicillin resistance; Nv^r^, novobiocin resistance; *larC*-encoding LarC (HVO_2381, UniProt D4GWM9); *glpK* (*hvo_1541*, UniProt D4GYI5); StrepII tag includes linker as follows: Gly-Thr-Trp-Ser-Pro-Gln-Phe-Glu-Lys; *pitA_Nph_*, *Natronomonas pharaonic pitA* (*np_2262*a, UniProt Q3IRM1) was used to replace *H. volcanii pitA* (*hvo_1871*, UniProt D4GSX4).

### Plasmid construction

To construct the plasmids listed in Table 1, high-fidelity, double-stranded DNA products were generated by polymerase chain reaction (PCR) using Phusion DNA polymerase (New England Biolabs). The primer pairs used for each PCR are listed in Table 2. *H. volcanii* genomic DNA, extracted as described in *The Halohandbook* (29), was used as the PCR template. All PCRs followed the manufacturer’s instructions, with 3% (vol/vol) DMSO added to the mix. PCRs were performed using an iCycler or MyCycler system (Bio-Rad Laboratories, Hercules, CA). A touchdown-PCR program was utilized to amplify the glycerol kinase gene, *glpK* (*hvo_1541,* UniProt D4GYI5). This program began with an initial denaturation at 98°C for 2 min, followed by two cycling blocks. The first block comprised 10 cycles of denaturation at 98°C for 10 s, annealing at 72°C for 30 s (decreasing 0.5°C per cycle), and extension at 72°C for 1 min. The second block included 25 cycles of denaturation at 98°C for 10 s, annealing at 67°C for 30 s, and extension at 72°C for 1 min. A final extension step at 72°C for 5 min concluded the reaction. DNA fragments were separated using 0.8% (wt/vol) agarose gels containing ethidium bromide (0.5 µg·mL⁻¹) in 1 × TAE electrophoresis buffer. Molecular weight standards (GeneRuler 1 kb Plus, Thermo Scientific, Waltham, MA) were used for size estimation. Gels were imaged with a Mini Visionary Imaging System (FOTODYNE, Hartland, WI). Before enzymatic modifications, PCR products were purified using the Monarch PCR and DNA Cleanup Kit (New England Biolabs). Plasmid DNA was isolated from *E. coli* strains using the PureLink Miniprep Kit (Invitrogen, Carlsbad, CA). Following the manufacturer’s protocols, inserts and vector DNA were digested with restriction enzymes (BlpI, NdeI, EcoRI, or XbaI). When necessary, digested plasmids were extracted from agarose gel slices using the Monarch DNA Gel Extraction Kit (New England Biolabs). Digested DNA fragments were ligated with T4 DNA ligase. Inverse PCR products were treated with DpnI and KLD Enzyme Mix per the supplier’s recommendations (New England Biolabs). The fidelity of all cloned PCR-amplified products was verified through Sanger automated DNA sequencing (Eurofins Genomics, Louisville, KY).

**TABLE 2:** Primers used in this study^*a*^

Primer	Sequence (5′-3′)	Description
F-*glpK*	GGTCATATGTCAGGAGAAACTTACGTCGG	HVO_1541 pJAM503; NdeI
R-*glpK*	TTTGCTCAGCTTATTCCTCCCGTGCCC	HVO_1541 pJAM503; BlpI
F-HvPrrn	CGATGCCCTTAAGTACAACAGGGT	pJAM-specific primers
R-T7Ter	AACCCCTCAAGACCCGTTTAGAG	pJAM-specific primers
NphpitA F	GAATTCATGCCACAACGCCAACCAC	*pitANph* forward primer; EcoRI
NphpitA R	TCTAGATCAGGCGAGGAAGACGTGG	*pitANph* reverse primer; XbaI
pitAF	GGAAAATCAAGCAGGTCATCGC	Forward primer specific to *pitAHvo*
pitAR	GTAGAACATCCCCATCGTGCC	Reverse primer specific to *pitAHvo*
NphPitAF	GCAGTATGCCGACAAGGTCTCC	Forward primer specific to *pitANph*
NphPitAR	CCCGCTCGTTTTTCCACAG	Reverse primer specific to *pitANph*

^*a*^ Restriction endonuclease sites used in cloning are underlined. F: forward. R: reverse.

### Construction of *H. volcanii* strains

To construct *H. volcanii* KM01, the *pitA* (*hvo_1871*, D4GSX4) gene of *H. volcanii* KS04 (H26 *ΔglpK*) was targeted for replacement with the *Natronomonas pharaonis pitA* (*pitA_Nph_*) gene using the *pyrE2*-based “pop-in/pop-out” method (30, 33). In this approach, KS04 was transformed with plasmid pTA1106 prepared in *E. coli* GM2163. *H. volcanii* transformants were plated on Hv-CA agar medium lacking uracil, and growth was counter-selected on media containing 5-FOA. The 5-FOA-resistant colonies were screened for the identification of strains with a desired replacement of the *H. volcanii pitA* with *pitA_Nph_*. PCR screening of colonies was performed using internal primers (*pitAF* and *pitAR*) specific to the target gene. Colonies that lacked the PCR product were further analyzed using primers (*NphPitAF* and *NphPitAR*), designed to anneal to the flanking regions. The resulting PCR products were resolved by gel electrophoresis and analyzed by DNA sequencing to ensure the accuracy of the KM01 strain.

### Complementation assay

The biological function of affinity-tagged GK variants was performed by complementation assay as previously described (13). The GK enzymes fused to an N-terminal polyhistidine tag (His-GK) and C-terminal StrepII tag (GK-StrepII) were compared. In brief, the parent H26, the mutant KS4 (H26 *ΔglpK*), and the mutant carrying either the plasmid expressing His-GK (KS4-pJAM4351), GK-StrepII (KS4-pJAM2666), or the empty vector (KS4-pJAM202c) were cultured on GlyMM and FMM plates (42°C, 5 days). Growth patterns were compared and imaged using an iBright Imaging System (Invitrogen). The data were interpreted based on the previous finding that *H. volcanii* requires GK when grown on GlyMM compared to FMM (13).

### Protein purification and analysis

*H. volcanii* H1207 strains expressing GK-StrepII (pJAM2666), His-GK (pJAM4351), or empty vector (pJAM202c) were used for protein purification. The *H. volcanii ΔglpK* mutant expressing His-GK (KS04-pJAM4351) was also used for purification. The strains were grown in GlyMM. Cells were grown to stationary phase (1.2 OD_600_, 1 OD_600_ unit equals approximately 10^9^ CFU·mL^−1^) in either 100 mL cultures in 500 mL flasks or 750 mL cultures in 2.8 L Fernbach flasks (200 rpm, 42°C, 5 days). Cells were harvested by centrifugation (25 min, 5,000 × *g*) at 21°C (room temperature, RT). Cell pellets were stored at −80°C until use.

For purification of GK-StrepII, cell pellets (from 750 mL cultures) were resuspended in lysis buffer: 20 mM Tris-HCl, pH 7.5, 2 M NaCl, 1 mM CaCl_2_, 3 mM MgCl_2_, 1 mM TCEP, 10 µg/mL DNase I, and EDTA-free protease inhibitor (1 tablet/10 mL). For each gram of wet cell pellet, 5 mL of lysis buffer was used. Cells were disrupted using a French pressure cell with four passes (20,000 lb∙in⁻², minimum high ratio of 140, Glen-Mills, NJ, USA). The lysate was centrifuged to remove debris (30 min, 13,000 × *g*, 4°C), and the supernatant was filtered sequentially through 0.45 µm and 0.22 µm pore-size filters. The GK-StrepII protein was purified using Strep-Tactin Superflow Plus Resin (binding capacity 9 mg protein/mL, U.S. Cat. No. 1057978 Qiagen, Germantown, MD). The resin (500 µL resin bed volume) was transferred as a 1 mL slurry to a 5 mL centrifuge column (U.S. Cat. No. 89897 Pierce, ThermoFisher Scientific) and equilibrated with binding buffer: 20 mM Tris-HCl, pH 7.5, 2 M NaCl, 1 mM TCEP. Clarified lysate was applied to the equilibrated resin, and the mixture was incubated at 4°C for 1 h with gentle rocking. After incubation, the column was centrifuged (500 × *g*, 1 min, RT), and the flow-through was collected. The resin was washed twice with 10-bed volumes of wash buffer: 20 mM Tris-HCl, pH 7.5, 2 M NaCl, 1 mM TCEP. Bound protein was eluted with two-bed volumes of elution buffer: 20 mM Tris-HCl, pH 7.5, 2 M NaCl, 1 mM TCEP, and 5 mM desthiobiotin. Elution was performed by rotating the resin at 4°C for 30 min, followed by centrifugation (500 × *g*, 1 min, RT).

For purification of His-GK, cell pellets (from 100 mL cultures) were resuspended in 1 mL of lysis buffer: 20 mM Tris-HCl or 50 mM HEPES, pH 7.5, 2 M NaCl, 5 mM β-mercaptoethanol, 40 mM imidazole, DNase, and EDTA-free protease inhibitor (1 tablet/10 mL). Cells were lysed in 1.8 mL microcentrifuge tubes by sonication on ice (four pulses of 10 s each) (Sonifier Cell Disruptor, Model W185, Heat Systems-Ultrasonics, Inc., Plainview, NY). The lysate was centrifuged (10 min, 13,000 × *g*, 4°C). The supernatant was applied to Ni-NTA His-Bind resin (100 µL slurry/50 μL bed volume, binding capacity 5–10 mg protein/mL; U.S. Cat. No. 70666-3 Millipore Sigma, Burlington, MA) preequilibrated in wash buffer: 20 mM Tris-HCl or 50 mM HEPES, pH 7.5, 2 M NaCl, and 40 mM imidazole. The mixture was incubated for 1 h at 4°C with gentle rocking. The resin was washed three times with 1 mL of wash buffer, and the His-GK protein was eluted three times with 100 µL of elution buffer: 20 mM Tris-HCl or 50 mM HEPES, pH 7.5, 2 M NaCl, and 250 mM imidazole.

### Protein quantification

Protein concentrations were measured using the Bradford assay (34) with bovine serum albumin (Bio-Rad Laboratories) as the standard. The assay was performed with the 96-well microplate format with a reaction volume of 250 µL. For each measurement, 5 µL of protein sample was mixed with 250 µL of Bradford reagent, and the mixture was incubated for 5 min at RT. Absorbance at 595 nm (A_595_) was recorded using a microplate reader (EPOCH2, BioTek, Agilent Technologies, Santa Clara, CA). The assay exhibited linearity within the 0 to 2 mg∙mL⁻¹ protein range.

### SDS-PAGE

Protein purity and subunit molecular mass were assessed by reducing SDS–PAGE with 12% polyacrylamide gels, run in Tris-glycine-SDS buffer at 120 V for 2 h. Protein fractions were prepared for electrophoresis by mixing an equal volume of Laemmli SDS sample buffer: 100 mM Tris-HCl (pH 6.8), 10% (vol/vol) β-mercaptoethanol, 2% (wt/vol) SDS, 10% (vol/vol) glycerol, and 0.6 mg/mL bromophenol blue. The samples were boiled for 10 min, chilled on ice for 5 min, and centrifuged at 13,000 × *g* for 10 min. Precision Plus Protein Kaleidoscope molecular mass marker (Bio-Rad Laboratories) was used as the standard. The gels were stained with Coomassie Blue and imaged using an iBright Imaging System (Invitrogen) according to the manufacturer’s protocol.

### Immunoblotting analysis

After SDS-PAGE, proteins were transferred to PVDF membranes (0.2 µm) (Amersham, Little Chalfont, UK) at 30 V for 14 h at 4°C with constant stirring as per standard protocol (Bio-Rad Laboratories). After protein transfer, the membranes were incubated with gentle rocking for 2 h at RT in blocking buffer composed of 5% (wt/vol) skim milk powder in TBST: 0.05 M Tris-HCl, pH 7.6, 0.15 M NaCl, 0.1% (vol/vol) Tween 20. To detect His-GK, HRP-conjugated 6 × His tag mouse monoclonal antibody (Proteintech Group, Inc., Rosemont, IL, U.S. Cat. No. HRP-660005) was diluted 1:10,000 in blocking buffer and incubated with the membrane for 1 h at RT. Following incubation, the membrane was washed five times with TBST for 5 min per wash. The Amersham ECL Prime substrate mixture (1:1) was applied to the membrane and incubated for 5 min prior to imaging with the iBright Imaging System (Invitrogen) according to the manufacturer’s protocol. To detect GK-StrepII, the rabbit anti-StrepII polyclonal “NWSHPQFEK” antibody (Genescript, Piscataway, NJ, U.S. Cat. No. A00626), diluted to 0.25 µg/µL in the blocking buffer, was incubated with the membrane for 1 h at RT. After washing the membrane five times with TBST, the secondary antibody, goat anti-rabbit HRP-conjugated (Southern Biotech, Birmingham, AL, U.S. Cat. No. 4010-05), was diluted 1:10,000 in blocking buffer and incubated with the membrane for 1 h at RT. Following secondary antibody incubation, the washing step was repeated. The Amersham ECL Prime substrate application and iBright Imaging were as described for His-GK.

### Size exclusion chromatography

Prior to size exclusion chromatography (SEC), protein samples were dialyzed using mini D-Tube Dialyzers with a molecular weight cutoff of 12–14 kDa according to the supplier (Novagen, Madison, WI). Samples were dialyzed (12–16 h, 4°C) against buffer (2 L per mL sample) composed of 50 mM HEPES, pH 7.5, 2 M NaCl, and 1 mM DTT with or without 10% (vol/vol) (1086 mM) glycerol. After dialysis, protein samples (100 µL per run) were applied to a Superdex 200 Increase 10/300 Gl (Sigma-Aldrich, St. Louis, MO, U.S. Cat. No. GE28-9909-44), pre-equilibrated in the dialysis buffer. The chromatography was performed in the dialysis buffer at a 0.3 mL·min⁻¹ flow rate. Molecular mass standards included vitamin B12 (1.35 kDa), myoglobin (horse, 17 kDa), ovalbumin (chicken, 44 kDa), γ-globulin (bovine, 158 kDa), and thyroglobulin (bovine, 670 kDa), and the void volume marker Blue Dextran (2,000 kDa) (Bio-Rad Laboratories). Protein elution was monitored by UV absorbance at 280 nm (A₂₈₀) and quantified using the Bradford assay (Bio-Rad Laboratories). Molecular mass estimations were derived from the linear regression (*R*² >0.99) of the logarithmic values of molecular mass against the gel phase distribution coefficient (K_av_). K_av_ was calculated using the equation: K_av_=(V_R_−V_o_)/(V_c_−V_o_), where V_R_ represents the retention (elution) volume of the protein, V_o_ is the void volume of the column, and V_c_ is the geometric bed volume.

### GK activity assay

GK activity was determined using a coupled assay in which the reduction of NAD^+^ to NADH was monitored by UV-Vis spectroscopy. In brief, GK catalyzes the ATP-dependent conversion of glycerol to glycerol-3-phosphate (G3P). This reaction is quenched, and then G3P dehydrogenase (G3PDH) is used to detect the oxidation of G3P to DHAP by monitoring the reduction of the electron acceptor NAD^+^ to NADH at absorbance 340 nm (A_340_). Purified His-GK was used for all activity assays. Reaction mixtures (1 mL) containing GK (0.84 µg protein, equivalent to 0.3 µM monomer), 3.5 mM MgCl₂·6H₂O, 3.5 mM ATP, 4.6 mM glycerol, 50 mM HEPES, pH 8.0, and 0.1 M NaCl were incubated at 57°C for 30 min unless otherwise indicated. After incubation, reaction mixtures (200 µL aliquots) were withdrawn and terminated by the addition of an equal volume of 0.2 N H₃PO₄. The samples were centrifuged at 12,000 × *g* for 10 min at RT to remove precipitated proteins. The second reaction was carried out as previously described (35). The G3P content of 110 µL portions was assayed enzymatically in a total reaction volume of 1 mL. The reaction contained 0.011 N NaOH for neutralization, followed by 1.1 mM NAD^+^, 0.66 M hydrazine sulfate (adjusted to pH 9.4 with NaOH), 1% (wt/vol) nicotinamide-sodium carbonate buffer, and rabbit muscle G3P dehydrogenase (8-10 U; Sigma, EC 1.1.1.8, 500 U). The reaction was incubated for 1 h at 30°C. NADH formation was measured at A_340_. Product formation was quantified using G3P standards ranging from 0 to 5 mM, demonstrating a linear relationship in assays (without GK), as confirmed by linear regression analysis (R² >0.99).

### GK activity optimization

The influence of pH, temperature, salinity, and ions (monovalent and divalent) on GK activity was tested by varying one parameter at a time while keeping others constant. The effect of magnesium concentration was tested across a range of 0–500 mM (0, 0.5, 3.5, 10, 20, 50, 100, and 500 mM MgCl_2_). In addition, the effect of different cations on GK activity was evaluated by comparing the influence of KCl, LiCl, CaCl₂, MnCl₂, ZnSO_4_, CoCl_2_, and MgCl₂ at a concentration of 3.5 mM. The effect of buffer and pH was tested using MES (pH 6.0, 6.5), HEPES (pH 7.0, 7.5, 8.0, 8.2), CAPS (pH 9.0, 9.5, 10.0), TES (pH 7.0, 7.5, 8.0, and 8.2), and Tris-HCl (pH 7.0, 7.5, 8.0, and 8.2) buffers. Temperature was tested from 7°C to 100°C (in 5°C increments up to 97°C, with a final test at 100°C). Salinity was tested from 0.1 to 4 M NaCl, with 0.1 M, 0.15 M, and 0.5 to 4 M NaCl concentrations in 0.5 M intervals. The salinity from the first reaction was diluted to a concentration that had no influence on the commercial G3PDH used in the second reaction (as confirmed experimentally). The effect of organic solvent (DMSO) was also tested at concentrations of 5% and 10% (vol/vol) DMSO with NaCl at 0.1, 1, 2, 3, and 4 M. For these experiments, the salinity carryover of the G3P product was equalized to 0.44 M in the second reaction, as this corresponded to the highest salinity condition tested.

### GK kinetic analysis and testing of fructose-1,6-bisphosphate as an allosteric effector

Kinetic analysis of GK was performed under the optimal conditions of pH (8.0), temperature (57°C), and salinity (100 mM NaCl). The concentration of each substrate and magnesium cofactor was varied to assess their impact on enzyme activity: magnesium (0, 0.05, 0.1, 0.15, 0.2, 0.3, 0.4, 0.5, 1.0, 2.0, and 3.5 mM MgCl_2_), glycerol (0, 0.2, 0.4, 0.6, 0.8, 1, 2, 3, and 4.6 mM), and ATP (0, 0.2, 0.4, 0.6, 0.8, 1, 2, and 3.5 mM). Kinetic parameters were compared using the Michaelis-Menten equation, Lineweaver-Burk plot, and Hill equation to analyze the enzyme’s behavior for each substrate and magnesium cofactor. Kinetic analysis was based on three experimental replicates with three technical replicates each. The potential inhibitory effect of fructose-1,6-bisphosphate (FBP), a known allosteric inhibitor of bacterial glycerol kinase, was evaluated using the *H. volcanii* GK. Enzymatic activity assays were performed in the presence and absence of 20 mM FBP in the reaction mixture to assess any changes in activity levels.

### Degradation of crude glycerol by His-GK

To evaluate the ability of *H. volcanii* GK to degrade crude glycerol, enzymatic activity assays were performed using crude glycerol as the substrate. The composition of the crude glycerol was determined by Eurofins Scientific Inc. Quality Trait Analysis. The sample contained 87% glycerol, 4.2% water, 4.5% ash, and 1.3% methanol. The amount of crude glycerol used in the assay corresponded to the glycerol concentration typically utilized in the activity assays, 4.6 mM. To reach this final glycerol concentration of 4.6 mM in the reaction mixture, the crude glycerol stock was diluted accordingly. This corresponds to approximately 0.386 µL of crude glycerol per mL of the reaction mixture. Based on this dilution, the final concentrations of the components in the reaction were as follows: 4.6 mM glycerol, 17.4 µg/mL ash, and 5 µM methanol. The enzymatic activity with crude glycerol was measured and compared to activity with pure glycerol under identical assay conditions to determine the relative degradation efficiency.

### GK tolerance to freeze-thaw, salinity, and temperatures

To examine the influence of freeze-thaw, the purified His-GK was dialyzed against 50 mM HEPES, pH 7.5, 2 M NaCl, and 1 mM DTT buffer containing 10% (vol/vol) glycerol. Enzymatic activity was measured from the freshly prepared protein. The sample was then stored at −80°C for 5 days. After storage, the sample was thawed, and the enzymatic activity assay was repeated to compare the GK activity before and after freezing. The stability of GK in different saline conditions was assessed by resuspending His-GK to a final concentration of 17 µg/mL in buffer containing 50 mM HEPES (pH 8) and NaCl at final concentrations of 0.1, 0.15, 1, 2, 3, and 4 M. The enzyme samples in the respective buffers were stored at 4°C, and enzymatic activity was measured at 0, 24, 48, and 72 h. For these enzymatic activity assays, the final GK concentration in each reaction was 0.84 µg/mL (equivalent to 0.3 µM homodimer). To isolate the effects of storage stability rather than salinity on enzymatic activity, the NaCl concentration in the first reaction at all conditions was standardized to 0.2 M by adding NaCl as needed. Temperature stability was tested under two salinity conditions: 0.1 M NaCl and 2 M NaCl. His-GK was diluted to 17 µg/mL in 50 mM HEPES (pH 8) with the respective salinity concentration and incubated at 24°C, 33°C, 42°C, 51°C, 57°C, 60°C, and 65°C for 1 h. After incubation, the enzymatic activity assay was performed under optimal conditions at 57°C. The final NaCl concentrations in the enzymatic reactions were 5 mM for the 0.1 M NaCl buffer and 100 mM for the 2 M NaCl buffer. A no-enzyme reaction was included as a blank for all measurements.

### Differential scanning fluorimetry thermal shift assay

His-GK was purified from KM01-pJAM4351 grown in GlyMM and stored at −80°C in a buffer containing 10% (vol/vol) glycerol. To assess the impact of ligand binding on protein stability, thermal shift assays were performed using various combinations of MgCl₂, ATP, and glycerol. Compared to the enzymatic assay (0.3  µM monomeric unit), the thermal shift assay used a 10-fold higher final enzyme concentration (3  µM), and ligand concentrations were scaled accordingly (35  mM MgCl₂, 46  mM glycerol, 35  mM ATP). To further evaluate the influence of glycerol under conditions consistent with those used in SEC analysis, DSF was used to compare the thermal stability of His-GK in the presence and absence of 10% (vol/vol) glycerol. The enzyme was diluted in a buffer containing 50 mM HEPES (pH 7.5), 2 M NaCl, and either 10% (vol/vol) glycerol or no glycerol, as indicated. All differential scanning fluorimetry (DSF) reactions were prepared in a total volume of 25 µL, and Sypro Orange Protein Gel Stain (5,000 × in DMSO, Invitrogen U.S. Cat. No. S6650) was added to a final concentration of 2.5×. Samples were blanked against a non-enzyme control containing the respective buffer condition to account for background fluorescence. The thermal shift assay was conducted using a C1000 thermal cycler with the CX96 real-time system (Bio-Rad Laboratories). Samples were subjected to a temperature gradient from 20°C to 95°C at a rate of 1°C per min. Fluorescence data were recorded continuously to monitor the unfolding process. The fluorescence intensity data were analyzed to identify the melting peak, which represents the temperature at which the protein unfolds. Results were compared across ligand combinations to evaluate their contribution to ligand-induced stabilization and between conditions with 10% glycerol and no glycerol to assess the effect of glycerol concentration on protein stability.

### Protein modeling

The 3D structural models of *H. volcanii* GK (UniProt ID: D4GYI5, HVO_1541) were generated using AlphaFold (36). High-confidence monomer and homodimer models were produced, with intrinsic predicted Template Modeling scores (ipTM) and predicted Template Modeling scores (pTM) of 0.94 and 0.92 for the monomer and 0.83 and 0.85 for the homodimer, respectively. To map the active site, glycerol was overlaid onto the AlphaFold-predicted models using structural alignment with the X-ray crystal structure of substrate-bound GK from *Thermococcus kodakarensis* (PDB ID: 6K79) as a reference. Ligands, including Mg^2+^, ATP, and glycerol, were positioned in the active site, and interactions were visualized using ball-and-stick diagrams. The AlphaFold-predicted Local Distance Difference Test (pLDDT) scores were used to assess the confidence of the monomer model, with a color gradient indicating per-residue reliability. The homodimer model was visualized with Coulombic surface coloring to illustrate electrostatic properties. Visualization and analysis were performed using ChimeraX (37). The N-terminal and C-terminal residues were annotated, and putative active site residues were identified and labeled based on UniProt annotations and spatial proximity to the ligands.

### Statistical analysis

Enzymatic activity assays were performed under defined conditions (as outlined in the earlier section). Each experiment included technical replicates performed in at least triplicate and was conducted a minimum of three times to ensure reproducibility. Data are presented as mean values ± standard deviation (SD). Statistical significance between experimental groups was assessed using Student’s t-test, with a threshold for significance set at *P*-value < 0.05. All statistical analyses were performed using Microsoft Excel.

## RESULTS

### 3D-structural modeling of *H. volcanii* GK

**Fig 1:**
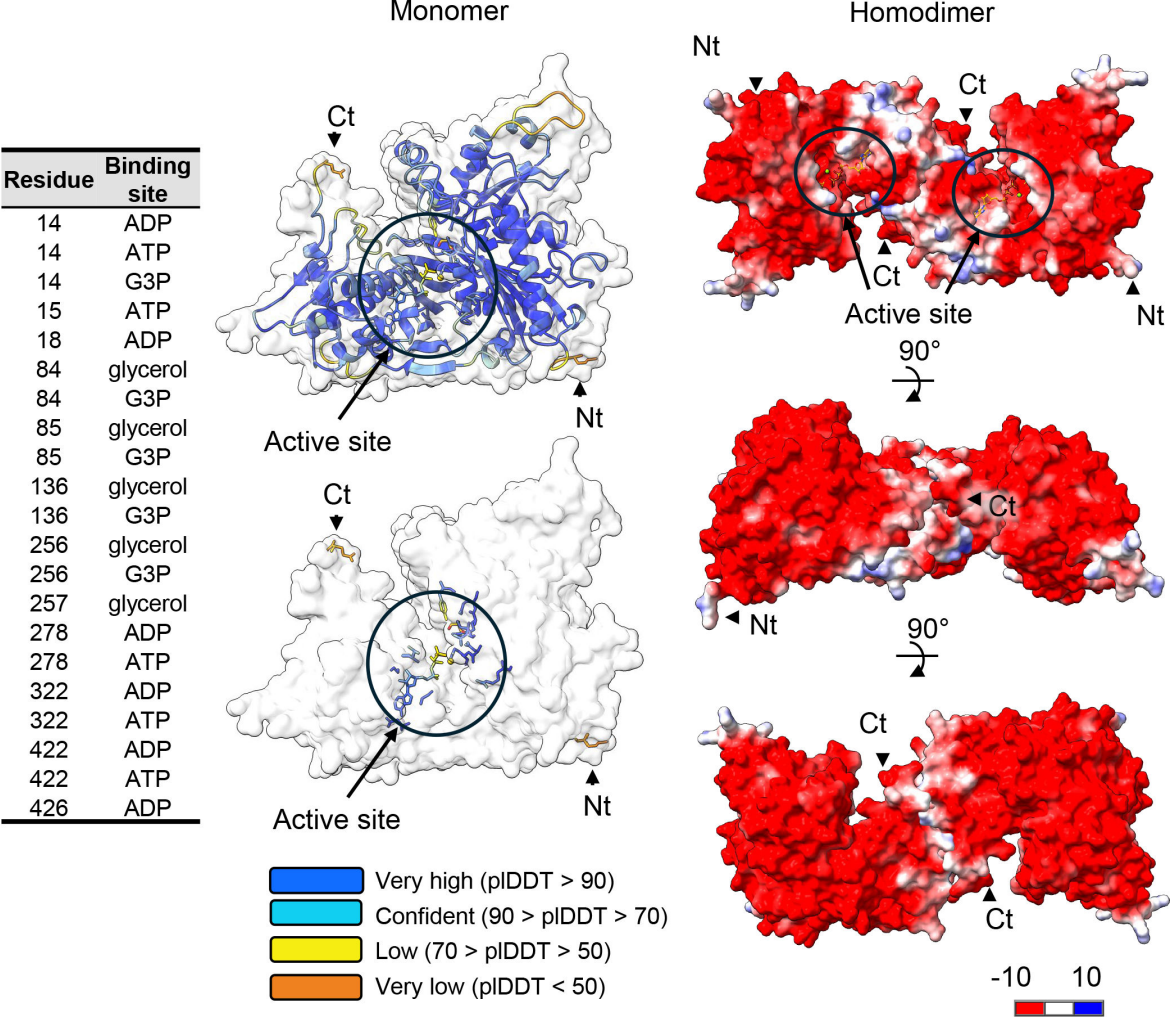
Three-dimensional structures of *H. volcanii* glycerol kinase (GK, HVO_1541 UniProt D4GYI5) bound to Mg^2+^ and ATP as a monomer (ipTM = 0.94 pTM =0.92) and homodimer (ipTM = 0.83 pTM =0.85) were modeled at high confidence using the AlphaFold 3 server. Other oligomeric configurations were predicted only at low confidence (ipTM <0.8). Glycerol was overlaid onto the 3D models using the X-ray crystal structure of substrate-bound GK of *Thermococcus kodakarensis* (PDB:6K79) as a guide. Active site ligands are represented in a ball and stick diagram. N-terminal (Nt) and C-terminal (Ct) residues are indicated. Coloring scales of monomer and homodimer indicated pLDDT as per-atom confidence and Coulombic surface coloring, respectively. Left list, residue number, and putative binding function based on UniProt annotation.

AlphaFold modeling was used to predict the 3D structure of the *H. volcanii* GK and determine the optimal location to attach an affinity tag to facilitate protein purification. The 3D modeling suggested the *H. volcanii* GK forms a homodimer and enabled prediction of the enzyme active site in this structure, including the binding sites for Mg^2+^, ATP, and glycerol (Fig. 1). The C-terminal region appeared near the active site and homodimer interface. By contrast, the N-terminal region was substantially distant from these locations. These results suggested that adding an affinity tag to the C-terminus may cause misfolding or obstruct the active site during purification, whereas tagging the N-terminal region was predicted to have minimal impact on the enzyme.

### Biological activity of affinity-tagged *H. volcanii* GK

**Fig 2:**
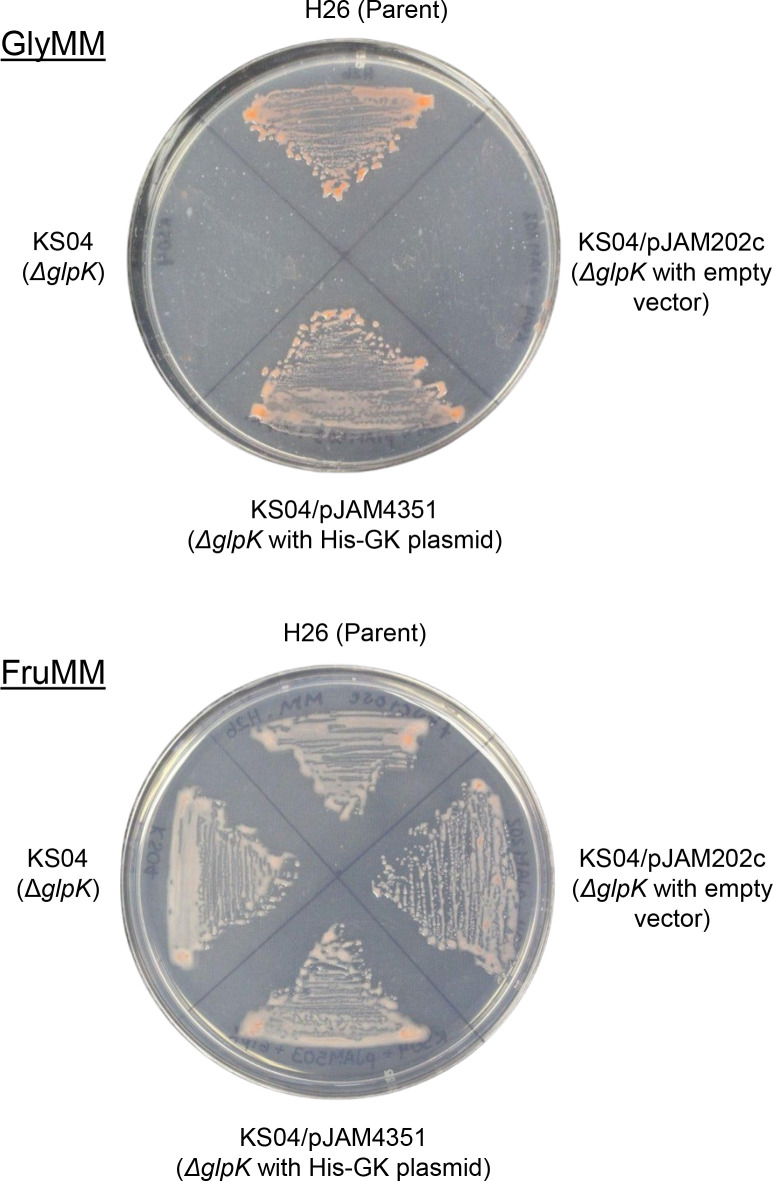
N-terminal His-tagged glycerol kinase is biologically active. *H. volcanii* strains were compared for growth on glycerol (GlyMM) and fructose (FMM) as the sole carbon source, as indicated. *H. volcanii* strains tested included: H26, parent; KS04, *ΔglpK* mutant; KS04/pJAM202c, *ΔglpK* mutant with empty vector; KS04/pJAM4351, *ΔglpK* mutant with His-GK expression plasmid. His-GK, N-terminal His-tagged glycerol kinase.

To further analyze the affinity tagging strategy, a complementation assay using a *ΔglpK* (GK) mutant strain was used. The *ΔglpK* (GK) mutant is unable to grow on glycerol unless the *glpK* gene is reintroduced to restore its activity or the cells are provided with an alternative carbon source like fructose (13). Our previous work demonstrated that GK fused to a C-terminal StrepII tag (GK-StrepII) retained *in vivo* functionality using this approach (13). Here, we examined the *ΔglpK* mutant expressing the GK fused to an N-terminal His tag (His-GK) from a plasmid. Through the expression of the His-GK, this strain was found to be restored for growth on glycerol to levels comparable to the parent (H26) (Fig. 2A)**.** By contrast, the *ΔglpK* mutant carrying the empty vector control was unable to grow on glycerol. As an additional control, all strains were shown to be able to grow on fructose (Fig. 2B). These results are consistent with the essential role of the GK in the metabolism of glycerol compared to other carbon sources and reveal that the N-terminal His-tag did not negatively impact the biological function of the *H. volcanii* GK.

### N-terminal His-tag is optimal for *H. volcanii* glycerol kinase purification

**Fig 3:**
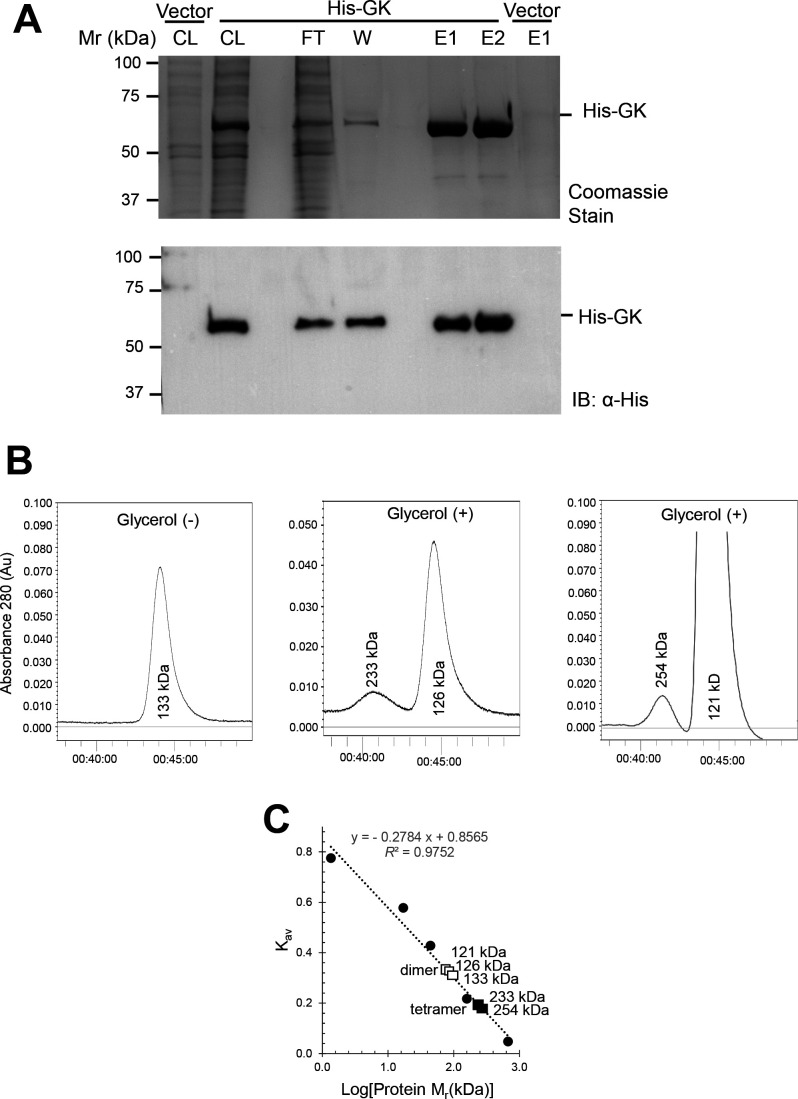
Purification of *H. volcanii* glycerol kinase (His-GK) and glycerol-induced shift from homodimer to homotetramer configuration. (**A) **His-GK is highly purified as demonstrated by SDS-PAGE. His-GK was synthesized in ATCC974-grown *H. volcanii* H1207-pJAM4351 and purified by Ni^2+^-NTA resin in 50 mM HEPES, pH 7.5, 2 M NaCl buffer. Vector, H1207-pJAM202c, with the empty vector control similarly analyzed for comparison. Protein fractions were separated by reducing 12% SDS-PAGE and analyzed by Coomassie stain and immunoblotting using anti-His antibodies (IB: α-His) as indicated. Cell lysate (CL), flow-through (FT), wash fraction (W), and elution fractions (E1 and E2, 2 µg per lane). Left: M_r_, molecular weight standards. (**B)** Glycerol-induced shift of His-GK from homodimer to homotetramer. His-GK expressed in GlyMM-grown *H. volcanii* H1207-pJAM4351, purified by Ni^2+^-NTA resin, and analyzed by SEC using a Superdex 200 Increase 10/300 Gl column in 50 mM HEPES pH 7.5, 2 M NaCl, 1 mM DTT supplemented with or without 10% (vol/vol) glycerol as indicated. Left: minus glycerol (Ni^2+^-NTA-purified sample). Middle: plus glycerol (Ni^2+^-NTA-purified sample). Right: plus glycerol (SEC-purified tetramer incubated overnight). (**C**) K_av_ vs. Log (Protein M_r_ [kDa]) plot. Molecular mass (M_r_) standards (closed circles), His-GK homodimer (open squares), and His-GK homotetramer (closed squares). M_r_ (*K*_av_) values observed for His-GK at 126 kDa (0.272), 133 kDa (0.265), and 233 kDa (0.197). Calculated theoretical M_r_ values for His-GK monomer (56.7 kDa), homodimer (113.4 kDa), and tetramer (226.8 kDa) as comparison.

To determine whether the N-terminal His-tag was optimal for GK purification, *H. volcanii* strain H1207 was used as host to express the His-GK from a plasmid (pJAM4351). The H1207 strain is genetically modified to avoid the non-specific binding of proteins to the Ni^2+^-affinity resin (31). After the growth of this expression strain in ATCC974-rich medium, the His-GK protein was purified by Ni^2+^-affinity resin. The GK-StrepII was similarly expressed in H1207 and purified by StrepTactin resin for comparison. By this approach, the His-GK was found to be of 4.5-fold higher purification yield (3 mg of protein per liter of culture or mg/L) than observed for GK-StrepII at 0.66 mg/L. Further analysis revealed His-GK was highly purified, as determined by SDS-PAGE followed by Coomassie blue staining and immunoblot analysis with anti-His antibodies (Fig. 3A). These results suggested that the use of the N-terminal His-tag would be ideal for *H. volcanii* GK biochemical studies.

### Oligomeric state of *H. volcanii* glycerol kinase affected by glycerol

To further characterize the biochemical properties of the enzyme, His-GK was purified from *H. volcanii* H1207-pJAM4351 grown on glycerol as the sole carbon source (GlyMM). Purification was done using Ni²^+^-affinity resin, followed by SEC to examine the structural conformation of the enzyme. In the absence of glycerol in the purification buffers, the SEC chromatogram showed a single peak corresponding to a GK homodimer (Fig. 3B). However, when 10% (vol/vol) glycerol was added to the buffers, two protein peaks were detected, with one corresponding to the homodimer and another to the homotetramer (Fig. 3B). To further examine these findings, the GlpK tetramer was isolated by SEC, incubated overnight at 4°C, and reanalyzed by SEC. After this reanalysis, the dimer was found to remain the predominant conformation of GlpK. Together, these results show His-GK exhibits a glycerol-dependent conformational shift from existing solely as a dimer to predominantly as a dimer with the additional appearance of a tetrameric form.

### Influence of metal ion, buffer, temperature, pH, and salinity on *H. volcanii* glycerol kinase enzymatic activity

**Fig 4:**
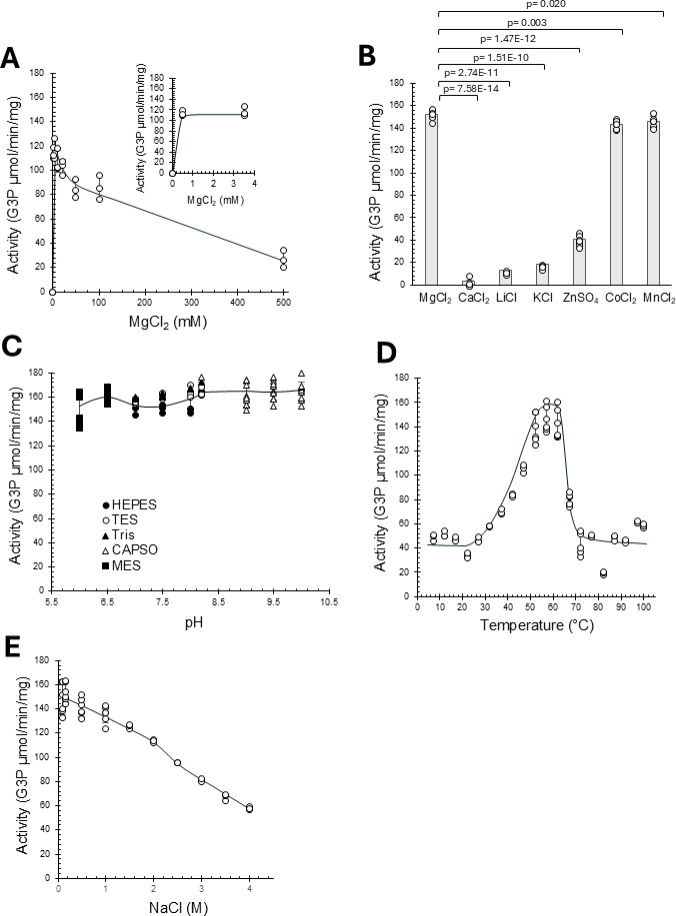
Optimal divalent cation, buffer, temperature, and NaCl conditions for *H. volcanii* glycerol kinase enzymatic activity. His-GK enzyme purified from *H. volcanii* KM01-pJAM4351 grown on GlyMM was used for activity assays. (A) His-GK activity in concentrations from 0 to 500 mM of MgCl_2_. (B) His-GK divalent metal ion requirement. Assay included 3.5 mM MgCl_2_ or replacement of this cationic salt with CaCl_2_, LiCl, KCl, ZnSO_4_, CoCl_2_, or MnCl_2_ as indicated. (C) His-GK activity from pH 6.0 to 10.0. Activity was analyzed with 50 mM HEPES, TES, Tris, MES, and CAPSO buffers as indicated. (D) His-GK activity at various temperatures. Activity was monitored in intervals of 5°C from 7°C to 97°C and at 100°C, at pH 8.0. (E) His-GK activity at different concentrations of NaCl. Activity was measured in 50 mM HEPES, pH 8.0, at 57°C with NaCl concentrations varied from 0.1 to 4 M NaCl. Statistical significance was evaluated using a t-test, with *P* < 0.05 considered significant.

To further investigate the biochemical properties and optimize the GK for biocatalysis, its enzymatic activity was assessed using a two-step reaction series. In the first step, glycerol and ATP were converted to G3P and ADP by GK. After quenching the reaction, the G3P product was diluted and quantified using G3P dehydrogenase, which oxidizes G3P to DHAP using NAD^+^ as the electron acceptor, which can be monitored spectrophotometrically. To evaluate the influence of reaction parameters on His-GK activity, one factor was varied at a time, while ensuring the second reaction in the series was not influenced by these alterations. As shown in Fig. 4A, His-GK required the addition of a divalent cation, such as Mg^2+^, for catalytic activity and demonstrated high activity across a wide MgCl_2_ concentration range (10–100 mM) with optimal activity from 0.5 to 3.5 mM MgCl_2_. Mn^2+^ and Co^2+^ could substitute for Mg^2+^ in this reaction (Fig. 4B). By contrast, the divalent cation Zn^2+^ only supported 30% of the activity, while the divalent cation Ca^2+^ and the monovalent cations K^+^ and Li^+^ were unable to serve as replacements (Fig. 4B). The activity of His-GK was relatively consistent across different buffer types (MES, TES, Tris, HEPES, and CAPSO) and a broad pH range (6.0–10.0) (Fig. 4C). By contrast, a clear temperature preference was observed, with optimal activity occurring at 50°C–60°C (Fig. 4D). Salinity also impacted activity, with the His-GK being more active at low concentrations (100–200 mM) of NaCl, yet still displaying ~50% activity at 4 M NaCl (Fig. 4E). With these variables optimized, His-GK was found to display an activity of 143 ± 17.0 µmol G3P/min/mg when assayed in 50 mM HEPES, pH 8.0, 100 mM NaCl buffer supplemented with 3.5 mM MgCl_2_ at 57°C.

### Kinetic parameters and positive cooperativity of *H. volcanii* glycerol kinase

**Fig 5:**
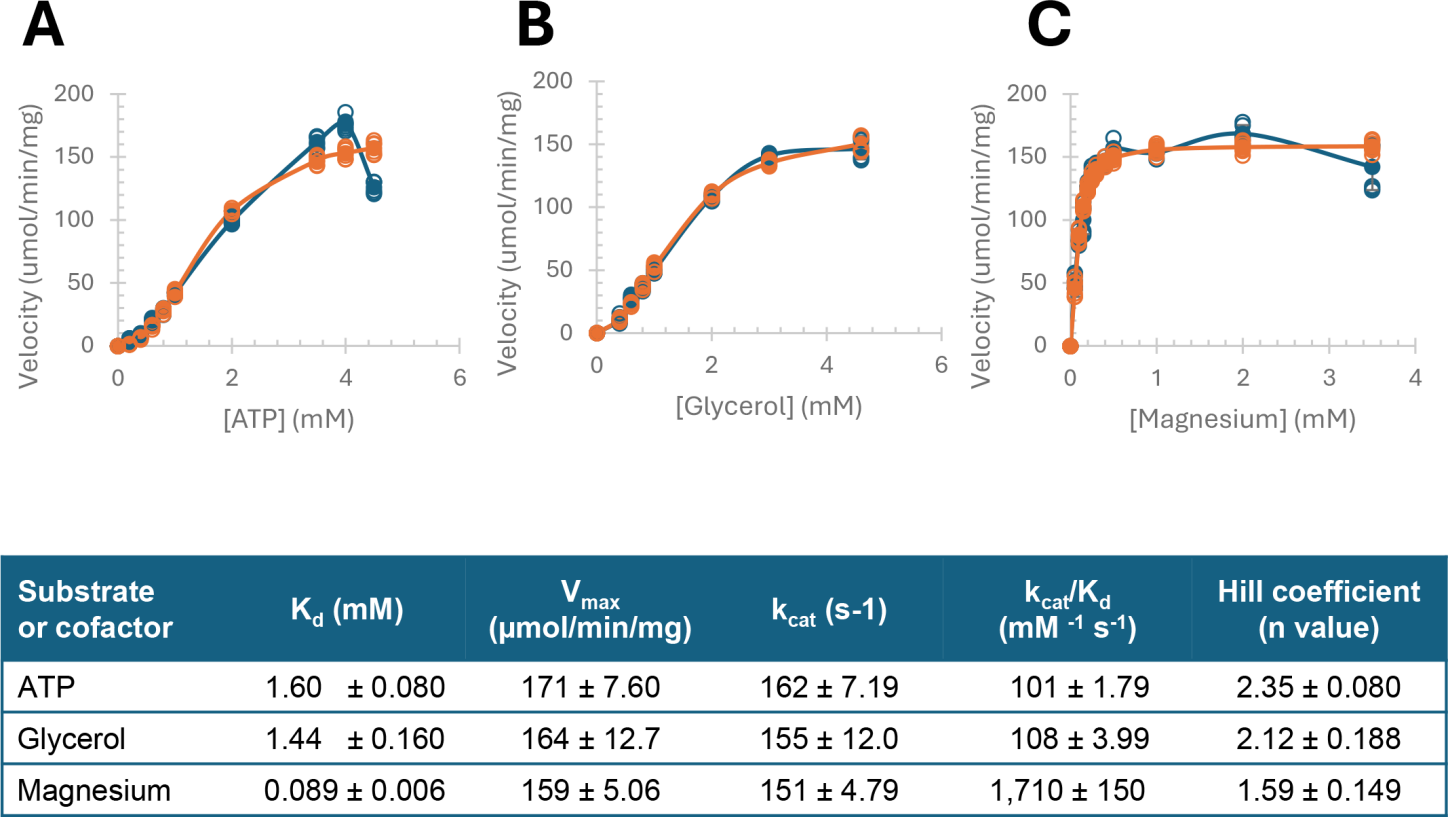
Kinetic analysis of His-GK with the substrates ATP and glycerol, and the cofactor magnesium. His-GK purified from KM01-pJAM4351 grown in GlyMM medium was used for all kinetic analysis. The activity assay was performed under optimal conditions using 50 mM HEPES buffer, pH 8, 0.1 M NaCl, and 57°C. The Michaelis-Menten equation describes a simple enzyme-substrate interaction as *v =* (*Vmax* [*S*])/(*Km +* [*S*]), where *v* is the reaction rate, *Vmax* is the maximum rate, [*S*] is the substrate concentration, and *Km* is the Michaelis constant. The enzymatic kinetics for GK exhibited non-Michaelis-Menten behavior, and data were analyzed with the Hill equation. Experimental velocity (blue) and calculated velocity (orange) are presented with ATP (**A**), glycerol (**B**), and magnesium (**C**) as indicated. GK activity was determined using a coupled spectrophotometric reaction with NADH production monitored by absorbance at 340 nm (A_340_). Kinetic analysis was calculated based on three experimental replicates with three technical replicates each. The Hill equation used was Y_calculated_ = (Vmax)[L]^*n* / (*K_d_* + [L]^*n*), where Y represents the fraction of occupied binding sites, [L] is the ligand concentration, *n* is the Hill coefficient indicating cooperativity, and *K_d_* is the dissociation constant. Squared residuals (Y_experimental_ − Y_calculated_)^2^ were summed to compute the sum of square residuals (SSR). Excel Solver was employed to minimize SSR and optimize the parameters for the best fit of the data.

Kinetic analysis revealed His-GK did not follow Michaelis-Menten behavior but instead exhibited positive cooperativity for ATP, glycerol, and magnesium, as indicated by Hill equation parameters (Fig. 5). The Hill coefficients (n) for ATP (*n* = 2.35 ± .08) and glycerol (*n* = 2.12 ± .19) were consistent with a strong positive cooperativity for the enzyme binding these substrates. The Hill coefficient for Mg^2+^ (*n* = 1.59 ± .15) suggested the cooperativity for this divalent metal cofactor was less pronounced compared to ATP and glycerol. Based on the sigmoidal behavior of the enzyme, dissociation constants (*K*_d_) were determined and found to be 1.6 ± 0.08 mM for ATP, 1.44 ± 0.16 mM for glycerol, and 0.089 ± 0.006 mM for Mg^2+^, suggesting the enzyme had a much higher affinity for the Mg^2+^ cofactor when compared to the glycerol and ATP substrates.

The maximum velocity (*V*_max_) and turnover number (*k*_cat_) values were comparable for all three reagents tested, averaging 165 ± 8.45 µmol/min/mg and 156 ± 7.99 s⁻¹, respectively. Thus, when the *K*_d_ values were considered to calculate the catalytic efficiency (*k*_cat_/*K*_d_), the substrates ATP and glycerol were found to be 16- to 17-fold lower at 101 ± 1.79 to 108 ± 3.99 mM⁻¹ s⁻¹, when compared to Mg^2+^ at 1,710 ± 150 mM⁻¹ s⁻¹. These results reveal that *H. volcanii* GK displays positive cooperativity with its substrates (glycerol and ATP) and has high catalytic efficiency with the magnesium cofactor, offering important perspectives on its enzymatic characteristics.

### Effect of organic solvent (DMSO), FBP, and crude glycerol on *H. volcanii* GK activity

**Fig 6:**
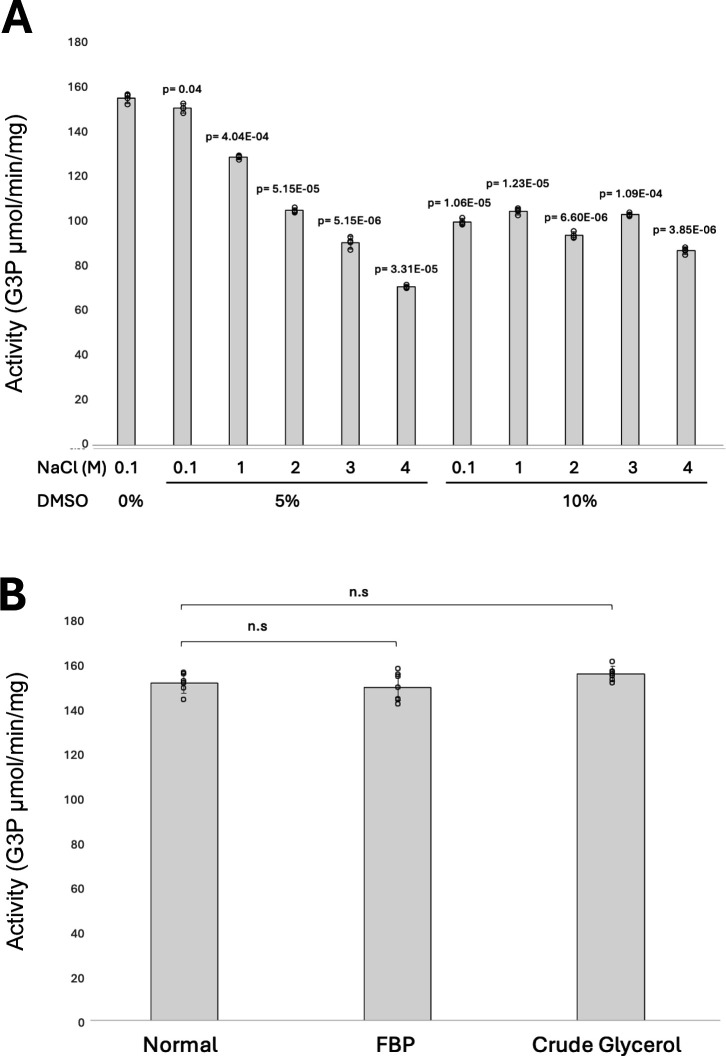
Effect of organic solvent (DMSO), FBP, and crude glycerol on *H. volcanii* GK activity. The His-GK enzyme, purified from *H. volcanii* KM01-pJAM4351 grown on GlyMM, was used for activity assays. (A) His-GK activity was evaluated in the presence of 5% and 10% (vol/vol) DMSO at NaCl concentrations of 0.1, 1, 2, 3, and 4 M. (B) The potential inhibitory effect of FBP was tested by adding 20 mM FBP directly to the reaction mixture. Enzymatic degradation of crude glycerol was performed using crude glycerol as the substrate. The enzymatic activity with FBP and crude glycerol was measured and compared to a control reaction where pure glycerol served as the substrate without FBP, under identical assay conditions, to determine the relative degradation efficiency. Statistical significance was evaluated using a t-test, with *P* < 0.05 considered significant and *P* > 0.05 denoted as not significant (n.s.).

The influence of organic solvents was assessed by measuring the catalytic activity of His-GK in the presence of 5% and 10% (vol/vol) DMSO at NaCl concentrations of 0.1, 1, 2, 3, and 4 M. As shown in Fig. 6A, the addition of 5% DMSO caused a gradual decline in enzyme activity as salinity increased. In 5% DMSO, the enzyme retained nearly full activity at 0.1 M NaCl but decreased to approximately half its activity at 4 M salinity. Although His-GK activity decreased at all salinities tested in the presence of 10% DMSO, the enzyme remained relatively active, maintaining an average of 63% activity regardless of salinity. To evaluate the potential of FBP as an allosteric inhibitor, His-GK activity was measured in the presence of 20 mM FBP, and the highest concentration reported to inhibit the *E. coli* GK (38). No significant inhibition was observed, as His-GK activity levels were comparable to the control reaction without FBP (Fig. 6B). In addition, the ability of His-GK to degrade crude glycerol, which contains the organic solvent methanol at a final concentration of 5 µM, was also tested. His-GK demonstrated the same efficiency in degrading crude glycerol as it did with pure glycerol (Fig. 6B).

### Stability of His-GK activity at varying temperatures and salinity levels

**Fig 7:**
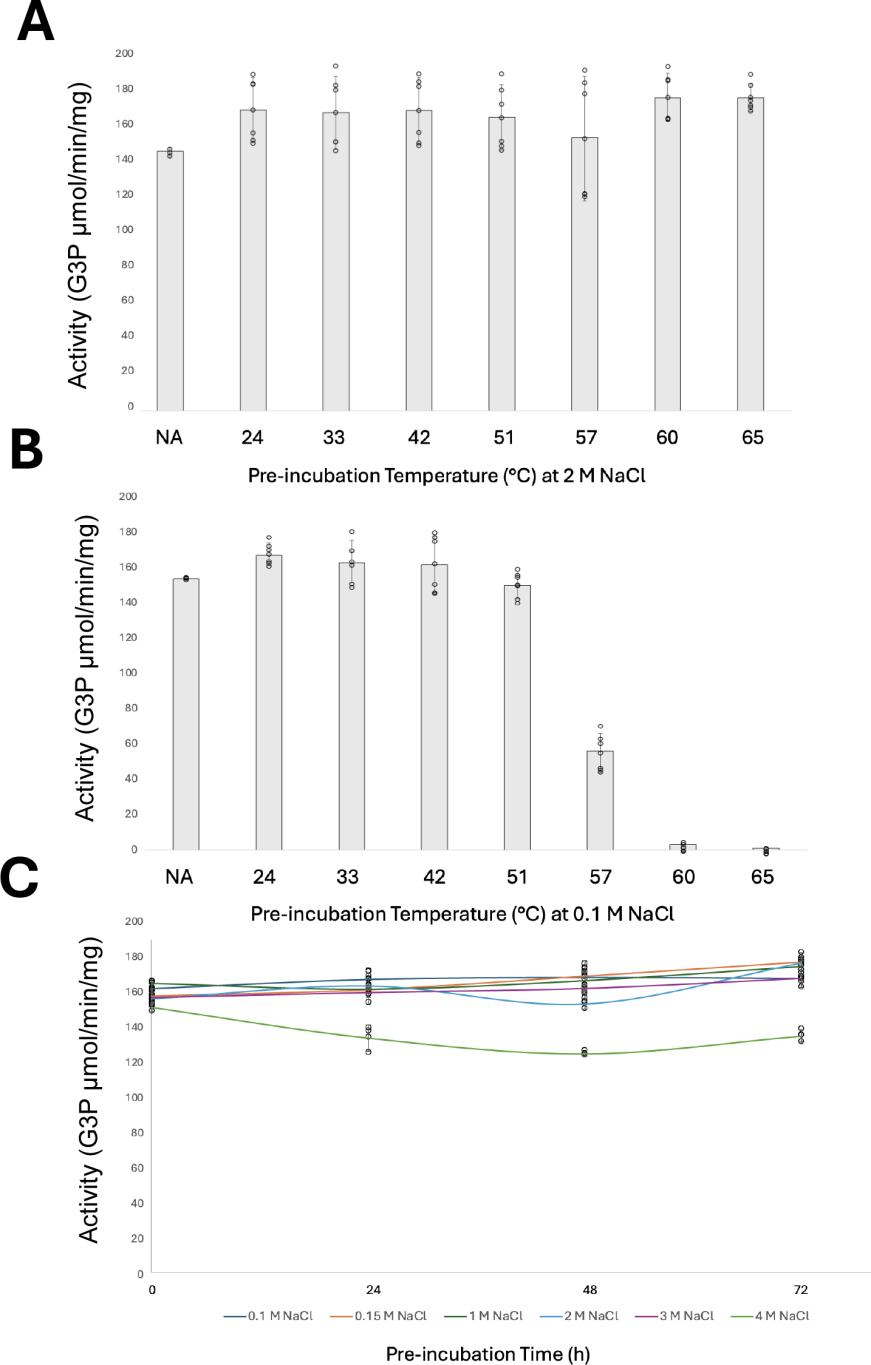
Stability of His-GK activity at varying temperatures and salinity levels. His-GK enzyme, purified from *H. volcanii* KM01-pJAM4351 grown on GlyMM, was used for activity assays. (A) His-GK activity was assessed after a 1 hour incubation at 24°C, 33°C, 42°C, 51°C, 57°C, 60°C, and 65°C in 50 mM HEPES buffer, pH 8, containing 2 M NaCl. (B) His-GK activity was assessed after a 1 hour incubation at the same temperatures in 50 mM HEPES buffer, pH 8, containing 0.1 M NaCl. (C) His-GK activity was measured after incubation in 50 mM HEPES buffer, pH 8, with NaCl concentrations of 0.1, 0.15, 1, 2, 3, and 4 M. Samples were stored at 4°C, and enzymatic activity was measured at 0, 24, 48, and 72 hours. NA represents the control reaction, where His-GK was directly assayed from the freezer without incubation.

To evaluate the stability of His-GK to freeze-thaw, the protein was stored in 50 mM HEPES, pH 7.5, 2M NaCl buffer containing 10% (vol/vol) glycerol at −80°C for 5 days. After thawing, enzymatic activity was reassessed. Remarkably, 100% of the enzymatic activity was retained after storage at −80°C, with slightly higher activity observed compared to the protein stored at 4°C. These findings indicate that storage at −80°C in glycerol-supplemented buffers effectively preserves His-GK activity, ensuring protein function and stability over time. To assess thermal stability, His-GK was incubated for 1 h at 24°C, 33°C, 42°C, 51°C, 57°C, 60°C, and 65°C in 50 mM HEPES buffer, pH 8, containing either 2 M NaCl (Fig. 7A) or 100 mM NaCl (Fig. 7B). Enzymatic activity was determined with the optimal parameters of 50 mM HEPES buffer, pH 8, and 57°C. The final NaCl concentrations were 100 or 5 mM, respectively. Remarkably, His-GK retained 100% of its enzymatic activity across all tested temperatures when incubated in the 2 M NaCl supplemented buffer (Fig. 7A). By contrast, under low salinity (100 mM NaCl), the enzyme remained stable up to 51°C but showed reduced activity at higher temperatures, retaining only 36% activity at 57°C. At 60°C and 65°C, the enzyme was fully inactivated (Fig. 7B). These findings suggest that long-term incubation at elevated temperatures requires high-salinity buffers to effectively preserve His-GK activity and ensure its stability over time. To evaluate His-GK stability under varying salinity conditions, enzymatic activity was measured after incubation in 50 mM HEPES buffer, pH 8, with NaCl concentrations of 0.1, 0.15, 1, 2, 3, and 4 M. Samples were stored at 4°C, and enzymatic activity was assessed at 0, 24, 48, and 72 h. Activity was reassessed with the optimal parameters of 50 mM HEPES buffer, pH 8, and 57°C, with a final reaction NaCl concentration of 0.2 M, ensuring all salinities were equalized during the enzymatic activity assay. The results demonstrated excellent stability across all tested salinities, with most conditions retaining 100% of enzymatic activity over time. The exception was 4 M NaCl, which showed a slight decrease in activity but still retained more than 80% activity after 72 h (Fig. 7C).

### His-GK thermal stability altered by ordered addition of ligands

**Fig 8:**
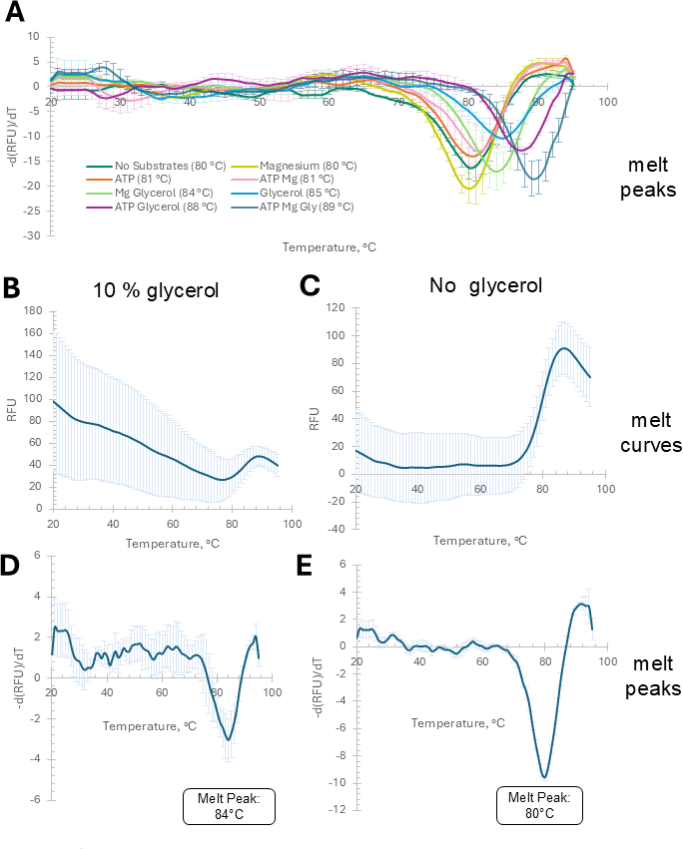
Thermal stability of *H. volcanii*. His-GK in the presence of ligands. His-GK, purified from *H. volcanii* KM01-pJAM4351 grown on GlyMM, was analyzed by thermal shift assay. The temperature was increased from 20°C to 95°C at a rate of 1°C per min. (A) Melt peaks of His-GK (3  µM) diluted in buffer containing 50  mM HEPES (pH 7.5), 2 M NaCl, and combinations of ATP (35 mM), MgCl₂ (35 mM), and glycerol (46 mM). B and C display melt curves, while D and E show the corresponding melt peaks. (B and D) His-GK diluted in buffer containing 50 mM HEPES, pH 7.5, 2 M NaCl, and 10% (vol/vol) (1068 mM) glycerol. (C and E). His-GK buffer was exchanged in the same buffer without glycerol. Glycerol alone increased T_m_ to 85°C, with further stabilization observed in combinations with MgCl₂ (84°C) or ATP (88°C). The highest T_m_ (89°C) occurred with all three ligands. MgCl₂ or ATP alone had minimal effect (80–81°C). Comparison of melt peaks in the presence of 10% glycerol vs. glycerol absence revealed a significant difference in T_m_ values (*P*-value = 0.004).

Thermal shift assays were conducted to evaluate the effect of ligand binding on the thermal stability of *H. volcanii* His-GK. In the absence of ligands, the enzyme exhibited a melting temperature (T_m_) of 80°C. The addition of MgCl₂, ATP, or both did not significantly alter the T_m_ (Fig. 8A; Fig. S1). By contrast, glycerol alone led to a marked increase in thermal stability, raising the T_m_ to 85°C (*P*  =  1.01  ×  10⁻⁴). Combinations of glycerol with ATP (88°C; *P*  =  7.3  ×  10⁻⁵) resulted in further stabilization. The highest T_m_ (89°C) was observed when all three ligands—glycerol, MgCl₂, and ATP—were present (*P*  =  8.0  ×  10⁻⁶), suggesting a cumulative stabilizing effect. To further investigate the influence of glycerol under conditions consistent with SEC analysis, DSF was used to compare melting profiles of His-GK in the presence and absence of 10% (vol/vol) glycerol. The melt curve patterns differed substantially depending on glycerol presence, with increased dye interaction at lower-to-intermediate temperatures (Fig. 8B and C). Glycerol significantly elevated the T_m_ from 80°C to 84°C (*P*  =  0.004), as determined from the first derivative of the melt curves (Fig. 8D and E). This glycerol-dependent shift in T_m_ is consistent with the enhanced thermostability of His-GK and correlates with its oligomeric shift from a strictly dimeric state to one where tetrameric forms also emerge, as previously observed via SEC.

## DISCUSSION

### *H. volcanii* GK displays biological function and can be highly purified when fused to an N-terminal poly-His tag

In this study, the *H. volcanii* GK was evaluated for the effects of fusing a poly-His tag to its N-terminus compared to adding a StrepII tag to its C-terminus. Both GK variants were found to be biologically active and capable of supporting the growth of *H. volcanii* on glycerol as the sole carbon source. By contrast, significant differences were observed in purification yield between the two proteins, with a 4.5-fold higher yield observed for His-GK compared to GK-StrepII. The 3 mg protein per L culture obtained for His-GK was useful for downstream biochemical analysis. SDS-PAGE confirmed high purity of the His-tagged GK, with only minor impurities. This outcome is consistent with the general trend that His-tagged proteins often achieve higher purification efficiencies than StrepII-tagged proteins (39). Expressing the protein in *H. volcanii* ensured proper folding, a common limitation when producing haloarchaeal proteins in mesophilic organisms such as *E. coli* (40, 41). Furthermore, the use of high-salt buffers likely minimized non-specific binding during purification (42). While both C-terminal and N-terminal tags have been used successfully, our 3D modeling suggests that the N-terminal His tag provides an optimal balance between functionality and purification efficiency for *H. volcanii* GK. The results emphasize the importance of tailoring tag selection and positioning to the specific characteristics of the target protein.

### Optimal conditions for different glycerol kinases

Glycerol kinases from various organisms exhibit distinct optimal conditions that reflect their environmental adaptations. In *H. volcanii*, the GK appears optimized for extreme conditions and can accommodate a variety of metal cofactors, including Mg^2+^, Mn^2+^, and Co^2+^. The enzyme’s catalytic activity operates over a wide pH range (6.0–10.0), is active in molar concentrations of salt, and functions at elevated temperatures, highlighting the adaptability of *H. volcanii* to hypersaline and thermophilic environments. Like the *H. volcanii* GK, many GKs use Mg^2+^ as the divalent metal cofactor (43, 44). However, most GKs display optimal activity at lower temperatures, are more sensitive to salt, and prefer neutral to higher pH values. For example, the *T. brucei* GK is optimal at pH 7–9.5 (45), while the human GK is best suited for a neutral environment with optimal activity at pH 7.5 (44). Other archaea, such as the hyperthermophilic *T. kodakarensis,* provide additional insight into GKs with unusual biochemical properties, as this enzyme prefers pH 8.0 and 80°C and favors cobalt ions (Co²^+^) over magnesium (Mg²^+^) for optimal activity (46). These findings emphasize that the choice of metal ion, pH, and other environmental factors significantly influence glycerol kinase activity across species. Enzymatic activity studies on *H. volcanii* GK further highlight how these environmental parameters—metal ion concentration, buffer type, temperature, pH, and salinity—shape its function.

### Effect of organic solvent and crude glycerol on *H. volcanii* GK activity

The organic solvent DMSO and crude glycerol, containing 5 µM methanol, on *H. volcanii* His-GK activity were also evaluated. These compounds have relevance to biotechnological applications in solvent-rich environments or crude feedstocks, where enzyme stability is essential. At 5% DMSO, His-GK activity decreased with increasing salinity, but still retained about half of its activity when assayed with 5% DMSO and 4 M NaCl compared to the no DMSO control at 0.1 M NaCl. This suggests that DMSO partially disrupts the enzyme’s activity at higher salinities. However, even at 10% DMSO, His-GK maintained about 63% of its activity across all salinity levels, indicating resilience to relatively high concentrations of DMSO in high-salinity conditions. Many halophilic enzymes are well known for their ability to perform in salt- and solvent-rich environments, which are common in industrial processes. For instance, *H. volcanii* alcohol dehydrogenase (ADH2) shows remarkable stability in the presence of DMSO and methanol (47), and *H. volcanii* laccase (LccA) retains over 50% of its activity after 24 h in DMSO (48). Similarly, the NAD^+^-dependent glutamate dehydrogenase from *Halobacterium salinarum* exhibits solvent tolerance (49). Moreover, His-GK demonstrated equivalent efficiency in degrading crude glycerol as it did with pure glycerol. This suggests that the enzyme is not only tolerant to methanol but also capable of efficiently catalyzing glycerol degradation in complex feedstocks. This robust enzymatic activity underscores its strong potential for industrial applications. When used in whole-cell systems, *H. volcanii* expressing His-GK may enhance the microbial conversion rates of crude glycerol in biodiesel production (2, 4, 5, 50). Beyond cellular applications, the purified enzyme itself holds promise for *in vitro* biocatalysis, where high salt and solvent tolerance are advantageous (7, 9). In addition, glycerol kinase’s specificity and activity make it a valuable candidate for biosensor development aimed at detecting glycerol, ATP, and divalent cation concentrations in industrial or clinical settings (7, 10, 11).

### Stability of *H. volcanii* His-GK activity at varying temperatures and salinity levels

The tolerance of *H. volcanii* His-GK to harsh environmental conditions was also evaluated. At 2 M NaCl, the enzyme retained full activity across all tested temperatures (24°C –65°C), showcasing its thermal stability under high-salinity conditions. By contrast, at 0.1 M NaCl, His-GK was stable only up to 51°C, with significant activity loss at 57°C and complete denaturation at 60°C and 65°C. These results underscore the critical role of salinity in preserving His-GK’s structural integrity and enzymatic activity at elevated temperatures. Interestingly, the thermal stability of *S. cerevisiae* mesophilic GK retains full activity up to 50°C after 1 h incubation and exhibits activity loss beyond this threshold (51).

### Glycerol-induced stability and oligomeric transitions of *H. volcanii* GK

Thermal shift assays revealed that *H. volcanii* His-GK exhibits high intrinsic thermal stability, with a T_m_ of 80°C under standard conditions. Ligand binding, particularly involving glycerol, significantly enhances the thermal stability of *H. volcanii* GK. No significant stabilization was observed with ATP, MgCl₂, or their combination, suggesting that these ligands alone are insufficient to promote structural changes detectable by thermal shift. By contrast, glycerol markedly increased the T_m_, both alone and in combination with other ligands. The additive effect seen with ATP and glycerol, and further with MgCl₂, suggests a cooperative stabilization mechanism, possibly reflecting sequential substrate binding. The addition of 10% glycerol increased the T_m_ to 84°C and introduced conformational heterogeneity in the melt profile, indicating dynamic structural transitions. These results suggest that glycerol promotes a more stable and potentially active conformation of the enzyme. Similar trends have been described in the thermophilic fungus *Chaetomium thermophilum* GK, where the T_m_ varies depending on the presence of glycerol and nucleotides (52). Interestingly, the T_m_ of *C. thermophilum* GK is reduced to 53°C–55°C in the presence of glycerol, ATP, or ADP, while increased when both nucleotide and glycerol are included in the assay, with T_m_ values reaching 63.6°C for the ATP-glycerol complex and 63°C for the ADP-glycerol complex (52), highlighting a ligand-dependent stabilization mechanism. *H. volcanii* GK appears to respond more robustly to glycerol, which may reflect its adaptation to high-salt environments. These findings support the hypothesis that ligand-induced oligomeric or conformational shifts contribute to enzyme stabilization and may inform future efforts in enzyme engineering.

In addition to enhancing thermostability, glycerol influenced the quaternary structure of His-GK. Size-exclusion chromatography showed that His-GK exists as a dimer in the absence of glycerol, but glycerol induces the appearance of a tetrameric form, suggesting changes in oligomeric state. This glycerol-dependent oligomeric transition mirrors regulatory mechanisms reported in other GKs. For example, *E. coli* GK transitions from an active dimer to an inactive tetramer in response to FBP (53). Likewise, the *Enterococcus casseliflavus* GK exhibits an active dimer conformation but forms a pseudo-tetramer under specific conditions (54). By comparison, GKs from *Plasmodium falciparum*, *Trypanosoma brucei gambiense*, and *C. thermophilum* maintain active dimeric conformations (52, 55, 56). Notably, the archaeal *T. kodakarensis* GK shifts from a dimer to a hexamer in the presence of glycerol. This conformational shift results in a closed structure that enhances ATP affinity tenfold, effectively regulating enzymatic activity (57).

These findings suggest that glycerol acts not only as a substrate but also as a modulator of His-GK stability and oligomeric architecture. Its stabilizing effect may result from reduced conformational flexibility and enhanced subunit-subunit interactions, facilitating the formation of a thermodynamically favored tetrameric state (58). This dual role of glycerol as a chemical stabilizer and a structural effector may reflect a physiological mechanism for optimizing GK activity under varying environmental conditions. Furthermore, understanding glycerol-mediated structural transitions could inform the development of robust halophilic enzymes for industrial applications, particularly in high-salt or solvent-rich biocatalytic processes.

### Positive cooperativity and kinetic parameters of *H. volcanii* GK

The glycerol-induced shift in the oligomeric state corresponds with our finding that *H. volcanii* GK exhibits sigmoidal kinetics and positive cooperativity for glycerol, ATP, and magnesium, as determined by the Hill equation. This finding suggests this enzyme is adapted to rapidly respond to variations in substrate levels. This dynamic behavior aligns with the organism’s adaptation to fluctuating environmental conditions and preference for glycerol over glucose. The Hill coefficients indicate strong cooperativity for ATP (*n* = 2.35 ± .080) and glycerol (*n* = 2.12 ± .188), with magnesium (*n* = 1.6 ± .15) showing a less pronounced cooperative interaction. The high affinity of magnesium (*K*_d_ = 89 ± 6 µM; *k*_cat_/*K*_d_ = 1,710 ± 150 mM⁻¹ s⁻¹) is consistent with other GKs used as biocatalysts in clinical and industrial settings, such as in the colorimetric detection of magnesium levels in serum (59).

*H. volcanii* GK exhibits lower affinity for ATP (*K*_d_ = 1.6 ± 0.08 mM) and glycerol (*K*_d_ = 1.44 ± 0.16 mM) compared to bacterial, eukaryotic, and other archaeal GKs. For example, the gram-positive bacterium *P. pentosaceus* GK has *K*_m_ values of 0.11 mM (glycerol) and 0.37 mM (ATP) (43), while *E. coli* GK has a *K*_m_ of 0.01 mM for glycerol (60). Eukaryotic GKs exhibit *K*_m_ values for ATP and glycerol in the range of 0.002–0.055 mM (61). Even the archaeal *T. kodakarensis* GK shows higher affinity (ATP *K*_m_ = 0.0154 mM; glycerol *K*_m_ = 0.111 mM) (46). These variations reflect the evolutionary divergence and environmental adaptations of GKs across domains of life.

The positive cooperativity of *H. volcanii* GK for glycerol, ATP, and magnesium is unique among GKs. This kinetic behavior aligns with our finding that *H. volcanii* GK undergoes a glycerol-induced transition from solely a dimer to a conformation where the dimer remains the predominant form, but a tetrameric form is also detected. This glycerol-induced shift in oligomeric state may enhance substrate affinity at other active sites. While this type of behavior has not been identified in other GKs, allosteric regulation is well-documented; for example, *E. coli* GK is inhibited by the allosteric effectors FBP and EIIA^Glc^, where FBP promotes a shift from a functional dimeric state to a less active tetrameric state (53, 62). In this mechanism, the bacterial GK exhibits positive cooperativity, meaning that the binding of one FBP molecule to the enzyme increases the affinity for subsequent FBP molecules to bind, leading to a more significant inhibition effect (63). These mechanisms in *E. coli* GK help regulate diauxic growth and prioritize glucose over glycerol via allosteric control, mainly by FBP (64). FBP does not appear to be a physiological regulator of eukaryotic GKs, where it causes a slight, if any, inhibitory effect on GKs from organisms such as the protozoan parasite *P. falciparum* (56), the thermophilic fungus *C. thermophilum* (52), and the salt-tolerant yeast *Debaryomyces hansenii* (65). Likewise, FBP is not an inhibitor of *H. volcanii* GK, consistent with its glycerol preference (13).

The cooperative kinetic profile of *H. volcanii* GK is a rare feature among glycerol kinases and suggests that the enzyme has evolved to function with high responsiveness to changes in substrate concentration. This ultrasensitive behavior has potential biotechnological relevance. Enzymes with such properties are highly valued in biosensing systems where a sharp transition between low and high activity states enhances signal clarity. Natural cooperativity, as demonstrated here, offers an advantage over synthetic tuning, as it simplifies sensor design and response calibration (66, 67).

Importantly, the biochemical resilience of *H. volcanii* in the presence of high salinity, elevated temperatures, organic solvents, and unrefined substrates suggests that it could withstand conditions that challenge most enzyme-based platforms. These characteristics make it a strong candidate for enzyme immobilization strategies such as cross-linked enzyme crystals (CLECs), which often demand tolerance to solvents and high ionic strength during processing (68). Few enzymes maintain activity under such extremes, making *H. volcanii* GK a unique tool for constructing reusable, stable biocatalysts.

In addition to its potential in glycerol biotransformation, *H. volcanii* GK could be repurposed for sensing biologically or environmentally relevant divalent cations. While magnesium is the canonical cofactor for GK and already forms the basis of clinical detection kits, the observed activity of this GK with manganese and cobalt indicates a broader substrate profile. This opens avenues for monitoring heavy metal contamination or metal homeostasis in complex samples (69).

*H. volcanii* GK’s environmental resilience and unique cooperative kinetics make it a strong candidate for both *in vivo* biocatalysis and *in vitro* biosensor development, especially under fluctuating conditions (9, 50). Its broad adaptability and substrate specificity present valuable opportunities for protein engineering in sustainable bioprocessing (70), including applications such as biodiesel waste valorization. Moreover, the use of isolated enzymes allows for tighter control over reaction conditions, enhanced product specificity, and greater compliance with regulatory standards (9–11). These advantages highlight the potential of *H. volcanii* GK for applications in diverse industrial and clinical settings—from diagnostic assays to environmental monitoring technologies. Our results, therefore, highlight the potential of GK not only in whole-cell systems but also in purified form for *in vitro* applications like biosensors or biocatalysis. Further structural studies aimed at uncovering the molecular basis of its positive cooperativity could support the rational design of improved GKs for industrial biocatalysis and selective detection of glycerol, ATP, and divalent cation concentrations in environmental, industrial, or clinical contexts.

### Conclusion

This study provides new insight into the functional characteristics of *H. volcanii* glycerol kinase, revealing that the enzyme not only tolerates harsh physicochemical conditions but also exhibits an uncommon degree of kinetic cooperativity. These findings suggest that *H. volcanii* GK is well-suited for future integration into modular biotechnological systems. Its native positive cooperativity enables sensitive and tunable responses to substrate levels, a property that could be harnessed in the development of biosensors requiring sharp activation thresholds. Moreover, the enzyme’s compatibility with multiple divalent cations and its structural resilience during exposure to solvents and high salinity make it a promising scaffold for further development. These properties support its potential in applications beyond glycerol metabolism—including CLEC-based immobilization strategies and real-time detection systems. Moving forward, *H. volcanii* GK could play a foundational role in building more adaptable and durable platforms for biosensing, biocatalysis, and environmental diagnostics.

## SUPPLEMENTAL MATERIAL

Fig. S1 aem.00886-25-s0001.tif Thermal shift profiles of *H. volcanii* GlpK.

ASM does not own the copyrights to Supplemental Material that may be linked to, or accessed through, an article. The authors have granted ASM a non-exclusive, world-wide license to publish the Supplemental Material files. Please contact the corresponding author directly for reuse.
