# Supplementary figures and images for: Biochemical properties of glycerol kinase from the hypersaline-adapted archaeon Haloferax volcanii
Source: Appl Environ Microbiol. 2025 Jul 8;91(8):e00886-25. doi: 10.1128/aem.00886-25 (PMC12366361; doi:10.1128/aem.00886-25)

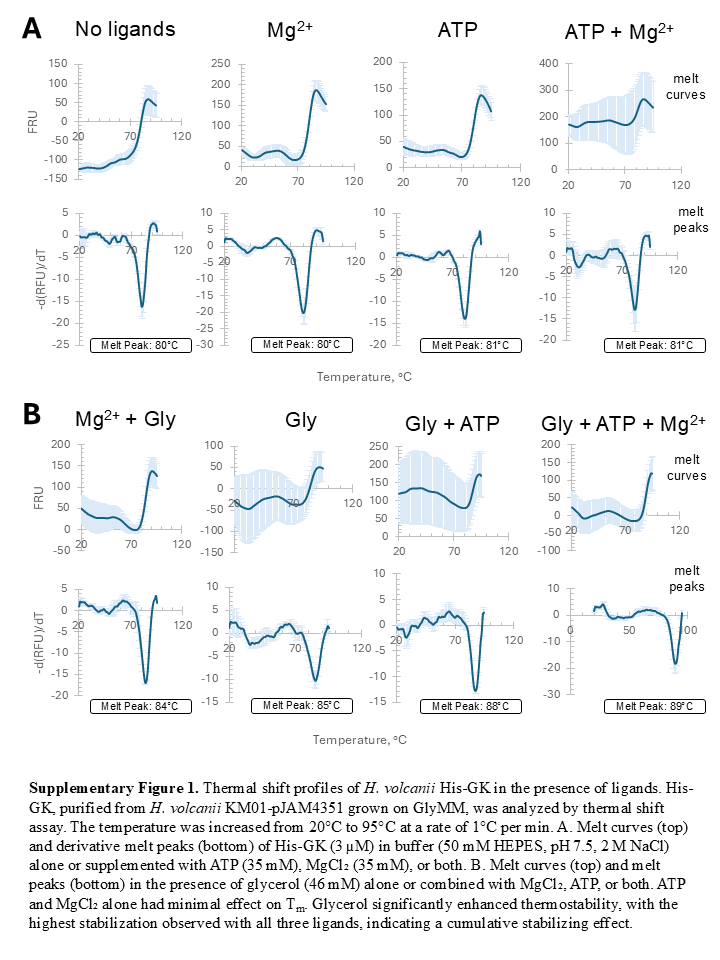

Supplement: Fig. S1 — Thermal shift profiles of H. volcanii GlpK. [file aem.00886-25-s0001.tif]
